# The mysteries of remote memory

**DOI:** 10.1098/rstb.2017.0029

**Published:** 2018-01-29

**Authors:** Zimbul Albo, Johannes Gräff

**Affiliations:** Laboratory of Neuroepigenetics, Brain Mind Institute, School of Life Sciences, Ecole Polytechnique Féderale Lausanne, CH-1015 Lausanne, Switzerland

**Keywords:** memory, consolidation, epigenetics, ACC, hippocampus, remote memory

## Abstract

Long-lasting memories form the basis of our identity as individuals and lie central in shaping future behaviours that guide survival. Surprisingly, however, our current knowledge of how such memories are stored in the brain and retrieved, as well as the dynamics of the circuits involved, remains scarce despite seminal technical and experimental breakthroughs in recent years. Traditionally, it has been proposed that, over time, information initially learnt in the hippocampus is stored in distributed cortical networks. This process—the standard theory of memory consolidation—would stabilize the newly encoded information into a lasting memory, become independent of the hippocampus, and remain essentially unmodifiable throughout the lifetime of the individual. In recent years, several pieces of evidence have started to challenge this view and indicate that long-lasting memories might already *ab ovo* be encoded, and subsequently stored in distributed cortical networks, akin to the multiple trace theory of memory consolidation. In this review, we summarize these recent findings and attempt to identify the biologically plausible mechanisms based on which a contextual memory becomes remote by integrating different levels of analysis: from neural circuits to cell ensembles across synaptic remodelling and epigenetic modifications. From these studies, remote memory formation and maintenance appear to occur through a multi-trace, dynamic and integrative cellular process ranging from the synapse to the nucleus, and represent an exciting field of research primed to change quickly as new experimental evidence emerges.

This article is part of a discussion meeting issue ‘Of mice and mental health: facilitating dialogue between basic and clinical neuroscientists’.

## 1. Introduction

Episodic memories are encoded within hippocampal and neocortical circuits [1] and can be retrieved long after they were initially allocated within the network [2–8]. Recent mnemonic information is thought to be dependent on an intact hippocampus [9,10], labile, readily amenable to disruption by *N*-methyl-d-aspartate (NMDA) receptor (NMDAR) antagonist or protein synthesis inhibitors [11,12], and more specific for details on contextual features [5,13]. On the other hand, remote memories, which we define here as memories lasting at least two weeks in rodents, are considered to be more stable or resilient to disruption as time passes by [14], independently retrievable by cortical entities such as the anterior cingulate cortex (ACC) for contextual memories, and more generalizable [15]. It is also believed that these remote mnemonic traces are shared by a distributed modality-specific (i.e. presenting a particular relationship between a stimulus-specific sensory modality pathway and behavioural performance) cortical network [16,17], and further maintained by several subcortical structures such as the basolateral amygdala [18–22] and mid-thalamic nuclei [23].

Within each structure of the network, formation, storage and retrieval of such a trace are likely contained in one and the same specific subpopulation of cells, named ‘engram' [24–33], which has certain characteristic features such as prime threshold activation by learning, the capacity to undergo plastic cellular and molecular changes, and the ability to get reactivated by a partial or incomplete stimulus [34–36]. However, with a few notable exceptions [18,37,38], what and where engrams are implicated in remote memory storage and how they change over time have received little experimental attention thus far.

Zooming into such memory traces, the synaptic storage of mnemonic information is thought to occur in basal and apical dendritic spines in pyramidal neurons: dendritic spines represent a means for structural remodelling in the brain where plastic changes occur during learning and memories get stored [39,40]. Notwithstanding, from engrams to spines surprisingly little evidence exists in the literature on the grounds of remote information processing, maintenance and storage to account for the lifelong and persistent nature of the mnemonic signal.

Inside neuronal cells, epigenetic mechanisms, i.e. ‘the structural adaptation of chromosomal regions so as to register, signal or perpetuate altered activity states' [41], might provide a nucleus-based solution to address some of these current issues and controversies, and to long-term memory storage in general [42–46]. Indeed, epigenetic mechanisms have for long been known to stably shape cellular identities throughout development, and can also readily react to changing environmental contingencies [47], but their importance in relation to across scale integration of learning processes from molecules to circuits is still in its infancy.

In this review, we uncover the still incomplete evidence on remote memory consolidation, from classic views to most recent ones, and attempt to understand how contextual information gets processed and stored in hippocampal–neocortical networks across different scales. Unless otherwise specified, we focus on contextual memories, as most of the literature on remote memories centres on contextual cues driving hippocampal–cortical interactions.

## 2. Memory consolidation and remote memory theories

The term memory consolidation was proposed more than 100 years ago by Müller & Pilzecker [48,49] and refers to a process by which new memories become gradually stabilized in order to persist for a long time. Historically, the hippocampus has been viewed as a temporary memory structure, while the cortex as one for long-term storage. The hippocampal formation is a three-layered structure—in contrast with the characteristic six layers of the neocortex—and comprises three distinct sub-regions: the dentate gyrus (DG), the hippocampus proper—also called Ammon's horn—consisting of CA3, CA2 and CA1, and the subiculum. Its deepest layer is rich in basal dendrites of principal cells while the most superficial layer contains the apical dendrites of the neurons and the large majority of axons that provide inputs. In terms of connectivity, the majority of hippocampal afferents originate from the entorhinal cortex via the perforant path and contralateral and ipsilateral hippocampal subfields. Contained within the sheets of cells is a functional trisynaptic circuit, oriented transverse to the main longitudinal septo-temporal axis, with entorhinal projections to dentate granule cells, granule cell projections onto CA3 pyramidal cells and CA3 pyramidal cell projections to area CA1, via the Schaffer collaterals [50].

**Figure 1.**
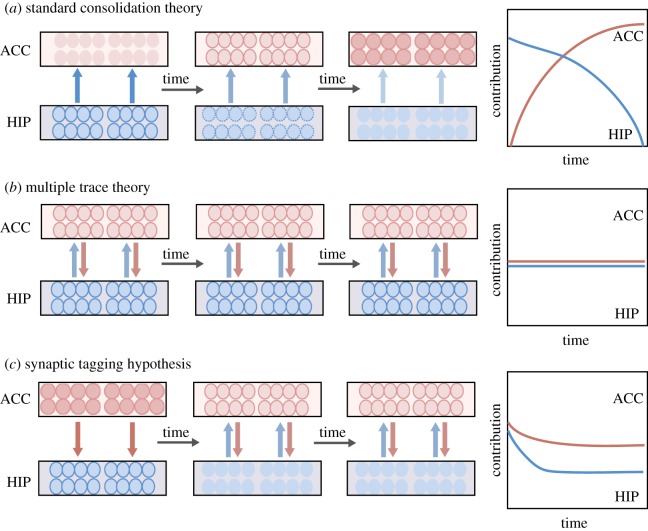
Current theories on remote memory formation and retrieval. (*a*) The standard consolidation theory states a linear relationship of decay in the hippocampus (HIP) and strengthening in the cortex, such as the ACC, over time, with the hippocampus unilaterally driving the mnemonic information transfer from earlier stages until the memory is completely transferred to cortical sites for its long-term storage. (*b*) The multiple trace theory postulates hippocampal–neocortical bidirectional interaction as early as the time of encoding as conjoint neuronal ensembles. Accordingly, the mnemonic trace is stored at multiple sites across the network, and for contextual or episodic memories, the influence of the hippocampus never decays. (*c*) According to the synaptic tagging hypothesis proposed here, an early distinctive synaptic or molecular signal occurs at the encoding in cortical sites and influences through as of yet unknown mechanisms the hippocampus for encoding. Such signal is critical for the formation of remote memories to persist over time. For references, please refer to the text.

According to the standard consolidation theory (figure 1*a*), a memory is initially hippocampus-dependent but, over time, undergoes a fortifying, i.e. consolidating, process and eventually becomes represented in a distributed cortical network independent of the hippocampus [51]. However, this classic view on consolidation is currently changing as more experimental evidence accumulates [52]. Consolidation is nowadays viewed as occurring both at the synapse and at the system level [8,53]: Synaptic consolidation refers to gene expression and synaptic changes occurring during the first minutes to hours after learning, whereas system consolidation spans a much broader, i.e. days to weeks, time scale until stability is achieved while transferring the mnemonic trace from hippocampus to cortex.

On top of that, another view on memory consolidation has gradually emerged, which refers to the distributed nature of long-term storage and which is known as multiple trace theory (MTT). MTT challenges some of the views of the standard consolidation theory as it attributes the hippocampus with a more enduring role throughout consolidation and retrieval, while conceiving the simultaneous importance of multi-site mnemonic traces across brain areas (figure 1*b*).

### (a) Synaptic consolidation

Synaptic consolidation refers to the cellular, molecular and synapse-based events neurons must undergo within the first few hours following learning to initially allocate unstable mnemonic traces into hippocampal circuits for later cortical and network consolidation. Although considered a relatively fast biological process, numerous studies report structural plastic changes in the form of dendritic spine formation and remodelling within 24 h [18,29,54–56]. Accordingly, synaptic consolidation is thought to be not only neuronal activity-dependent, but also gene- and protein synthesis-dependent. Among the best characterized changes for synaptic consolidation are the activation of CREB (cAMP response element binding protein)-dependent gene expression changes [57–59] as well as the translation, at activated synapses, of the immediate early gene (IEG) *Arc* (activity-regulated cytoskeletal protein), believed to play a key role in actin cytoskeletal dynamics and to regulate the membrane expression of various postsynaptic receptors [60,61]. In addition to such cytosolic plasticity-related proteins, dendritic mRNAs have also been proposed as diffusible plasticity-related molecules that may underlie synaptic consolidation [62]. The long-term synaptic plasticity associated with these early changes is then also accompanied by structural changes at synapses, which involve, among other processes, actin polymerization [63,64] and the p21 kinase-activated cofilin cascade, which promotes cytoskeleton assembly and regulates spine morphology [63,65–67].

Because of the inherent short time scale of the abovementioned changes, synaptic consolidation as a first step towards the formation of mnemonic traces cannot, however, account *per se* for the extended dynamics, stability and persistence required for truly long-lasting memories. For instance, synaptic plasticity itself, such as long-term potentiation (LTP) is classically known to be responsible for the learning of new associations and spatial features [68–71], but its role in remote storage is less clear [72,73]. In this regard, the synaptic tagging and capture hypothesis [74], which essentially states that tagged synapses (which are defined as short-lived targets of unknown molecular identity, important for subsequent neural plasticity, and previously induced by activity-dependent processes during learning and memory) can capture plasticity-related proteins that stabilize synaptic modifications [62], offers an alternative. For instance, it has been proposed that under strong tetanization, a given synaptic pathway can undergo a local tag setting with the synthesis of diffusible plasticity-related proteins that are then captured by tagged synapses, a necessity for the maintenance of late long-term potentiation (L-LTP), which itself is a pre-step towards enduring memories [71,75].

In a related set of ideas regarding synaptic tagging but with more emphasis towards remote memory circuits and behaviour, an interesting study using c-Fos imaging and local pharmacological inactivation proposed that early tagging of cortex during memory encoding is required for the formation of enduring associative memories that support remote memory storage [76]. Accordingly, synaptic and cellular tagging mechanisms could generate an activating and strengthening signal in relevant distributed cortical cell assemblies over time, favouring a post-learning mechanism underlying systems-level memory consolidation. In this study, the social transmission of food preference (STFP) task, a hippocampus-dependent ethologically based variant of associative olfactory memory, was used to show early involvement of the orbitofrontal cortex (OFC), a critical site for remote storage of this type of memory. Remote memory formation was impaired when hippocampal activity was pharmacologically silenced during the early (1–12 days), but not the late (15–27 days), post-learning period. Unexpectedly, however, silencing neuronal activity in the OFC early post-learning also impaired remote memory and structural plasticity, indicating that early cortical activity is required for subsequent maturation and stabilization of the mnemonic traces. Such early tagging in the OFC was found to be NMDAR-dependent and to trigger signalling cascades leading to histone acetylation, an epigenetic modification. Intriguingly, the engagement of the OFC was odour-specific, which suggests that tagging may minimize interference during the consolidation process, for instance by making the new trace more compatible with existing cortical mental schemas [77,78]. Thus, this new variant of synaptic tagging and capture (figure 1*c*) attractively shows how local molecular changes may mark synaptic plasticity in circuits implicated in remote memory consolidation [79], but awaits further confirmation for other types of memories.

### (b) Standard system consolidation theory

Moving beyond the focus on local synaptic processes to a more systems level, the standard consolidation model proposes that long-term memory encoding involves early plasticity within hippocampal circuits, whereas reorganization of the neocortex is required weeks to months later to subserve remote memory storage (figure 1*a*). The hippocampus is thus thought to be of time-limited importance and only initially needed for storage and recovery of a memory [9,80]: as memories mature they become increasingly dependent on the cortex in a ‘mnemonic shift' or ‘information transfer' from the hippocampus [7]. Lesions of the medial temporal lobe (MTL), accordingly, would lead to a retrograde amnesia for pre-lesion events, with a temporal gradient observed for long-term episodic memories.

Indeed, much past and present emphasis on temporal lobe localization of memory function can be attributed to the clinical and cognitive characterization of the profound anterograde and temporally graded retrograde amnesia observed in patient H.M., after bilateral surgical resection of most of his MTL was performed in an attempt to ameliorate his suffering from intractable epilepsy ([81], for an extended review, see [82]). Although there is no prediction about the involvement of different MTL structures in remote memory nor the relationship between lesion size and remote memory loss, numerous other studies also testify to the reversible inactivation of the ACC as being disruptive to remote memories without affecting recent ones [6,7,14,83,84].

Yet, as several studies on NMDAR functioning—critical for synaptic plasticity as well as learning and memory [85]—exemplify, there appears to be a need for an update on this theory as first, the hippocampus might also be required for remote memory storage, and second, the ACC might also be needed for recent memory formation. For the hippocampus, ibotenic hippocampal lesions immediately after conditioning (but not 24 days later) were found to not only prevent dendritic spine growth in the ACC, but also to impair remote contextual fear memory [55]. Similarly, intact hippocampal NMDAR function was found to be necessary not only for recent, but also for remote contextual fear and spatial memory consolidation [85]. For the cortex, cingulate NMDAR function was not only critical for the induction of local LTP, but its pharmacological or genetic blockade also impaired the formation of early contextual fear memory [86]. Furthermore, divergent patterns for NMDAR blockade in adult neocortical and hippocampal pyramidal neurons have been speculated as forming the basis for differential spine regulation and turnover dynamics in both structures [54], further lending support that there might be alternative explanations as to how remote contextual memories are formed and stored [52].

### (c) Multiple trace theory (distributed cortical–hippocampal network for remote memory)

An alternative view to the standard consolidation theory is the multiple trace theory (MTT). MTT [17,87,88] states that the hippocampus is needed for re-experiencing detailed recent or remote episodic memory, contributes to formation of cognitive schemas [89], and postulates that long-term storage of information occurs in a distributed cortical network [90] where different modality-specific ‘fragments of a memory' co-exist across different sensory cortices (figure 1*b*). According to MTT, each time an episodic memory is retrieved it is subsequently re-encoded, thereby leading to the formation of multiple traces mediated by ensembles of hippocampal–neocortical neurons [89]. MTT has thus three components [16]: (1) an initially formed memory remains dependent on the hippocampus for as long as it is available; (2) a hippocampal memory over time supports the development of a less integrated or schematic version of the memory in the neocortex (retaining the gist of the original memory, but fewer contextual details); (3) a dynamic interplay exists between the cortical and the hippocampal versions of the memory such that one or the other may be dominant depending on the circumstances at retrieval.

While the precise demonstration of each criterion in a single study remains elusive, several experiments nevertheless provide strong evidence in support of MTT. Using optogenetic inhibition or local lesion studies in prefrontal cortical areas, several studies independently showed an early involvement of the prefrontal cortex in remote contextual fear memories [18,91,92]. Likewise a pervasive involvement of the ACC in memory encoding was reported through the use of NR2B antagonists and protein synthesis inhibitors [83]. Conversely, in studies focusing on the persistent involvement of the hippocampus, the need for an intact hippocampus to avoid deterioration of remote memories was reported using excitotoxic lesions [4], optogenetically [2], and by using protein synthesis inhibitors [93] at stages when the hippocampus was assumed to be no longer required, namely following remote memory recall. This last study sparked considerable interest as it showed that even during remote memory stages mnemonic information can enter a period of lability akin to the initial consolidation phase itself, coined ‘reconsolidation' (for comprehensive reviews on this topic, see [94–98]).

Interestingly, mnemonic neural ensembles have also been identified outside of the medial prefontal cortex (mPFC), namely in retrosplenial cortex [99], where optogenetic stimulation was reported sufficient to produce both context-specific memory changes. Finally, in an impressive brain-wide imaging study, post-training chemogenetic silencing of previously identified high-degree memory nodes, which are important for long-term memory storage based on the analysis of IEG expression patterns [100] and belong to neither the hippocampus nor the ACC, were also found to disrupt remote fear memory consolidation [101]. Together with a study showing remote memory consolidation deficits upon systemic administration of anisomycin, but not of local infusions into the ACC [102], these studies emphasize a more widespread nature of the mnemonic signal across time and the brain than previously anticipated.

## 3. Engrams in remote memory

As mnemonic traces seem to be of a more distributed nature, the next logic step is to locate these traces within the engaged brain structures. Mnemonic ‘engrams' were proposed more than a century ago as the physical substrate of a memory within the brain [103,104] and owing to recently developed genetic tools have lately become available for visualization and activity manipulations [24,105–107]. An engram is conceived as an ensemble or population of activated (at encoding, by learning or conditioning) excitatory neurons in brain structures of mnemonic circuits designed to retain over time (with enduring plastic cellular changes) learned associations encountered in the environment and capable of being reactivated by a part of the original stimulus for recall [36]. Although several elegant studies have testified to the importance of engrams in memory encoding and recent memory storage [25,28–32,108,109], most of them, for technical reasons, focused on the DG as opposed to CA1, although it is hippocampal area CA1 that has traditionally received more attention in the field of episodic memory research [110–112]. And, only few studies have attempted to address the formation and maintenance of remote engrams.

Among them, Tayler *et al.* labelled neurons with human histone H2B-GFP driven by a doxycycline-inducible IEG c-Fos promoter in a TetTag double transgenic mouse system (H2B-GFP TetTag mice) during a contextual fear conditioning task [37]. They found a large network of tagged neurons in hippocampus, amygdala and neocortex, which was activated upon recent retrieval, but after two weeks the pattern of activation of the ensemble only persisted in cortex. These findings naturally favoured the classic view of temporal consolidation but incorporated also features of MTT as multiple sites contained mnemonic information.

In another study Denny *et al.* designed a tamoxifen-inducible ArcCreER**^T2^** transgenic mouse line to compare encoding and expression at recent and remote timepoints in the hippocampus [38]. This ArcCreER mouse line is similar to the TetTag mouse line mentioned above but based on the IEG Arc promoter to access the engram population, and on tamoxifen to restrict such access to specific timepoints. They found a greater than chance percentage of reactivated cells in the DG and also in area CA3 for recent memories. However, over time (30 days) animals generalized between contexts and both reactivation rates decreased, which could be interpreted as hippocampal memories being redistributed to cortical sites. However, when encoding neurons in DG or CA3 were optogenetically silenced using the Arch-GFP line, memory retrieval was impaired, which highlights the necessity of the original ensemble's lasting activation for fear expression. Since in mice with reduced neurogenesis—mediated, in part, by the DG [113,114]—contextual fear memory appeared to be less precise and the degree of reactivation in CA3 (but not DG) was reduced, the authors concluded that the degree of CA3 activation (but not DG) was related to the strength of the memory trace, despite the acknowledged importance of engram cells within the DG in contextual fear memories [29–32,108]

Finally, in the most recent study regarding remote memory consolidation, Kitamura *et al.* used a series of state-of-the-art optogenetic and calcium imaging tools and found that neocortical prefrontal memory engram neurons were already generated rapidly during initial conditioning through inputs from both the hippocampal–entorhinal system and the basolateral amygdala [18]. With time, these prefrontal engram cells became functionally mature, whereas hippocampal engram cells gradually became silent. Although this study did not assess activity-dependent labelling of prefrontal engrams for direct comparisons with hippocampal ensembles, this is the first report of its kind to probe the hippocampal–mPFC circuitry during long-term memory consolidation in an engram-specific manner. Interestingly, these findings combine elements of both the standard and the multiple trace theory of system consolidation, and thus await further confirmation in other studies.

## 4. Long-term memory and dendritic spines

The question, nevertheless, remains, within engram cells, but also more generally speaking, what are the neuronal structures that store a memory? Dendritic spines represent the postsynaptic component of excitatory synapses and their growth has been postulated as a necessary mechanism of neural circuits to accommodate plastic changes taking place during a learning-engaged signalling cascade. The size and density of spines during this structural remodelling process have been found to change in a number of synaptic and behavioural plasticity paradigms, leading to the suggestion that they may form a structural basis for long-term memory [39,115–119]. Dendritic spines show different shapes: mushroom, thin, stubby and branched types, while the types most reported in memory studies are the thin spines with a small head (i.e. *thin spines*), thought to be highly plastic due to their underlying experience-dependent rewiring capacity, and the spines with a large, mushroom-like head (i.e. *mushroom spines*), which are considered more stable and to represent the physical substrates of long-term memories [40,120].

**Figure 2.**
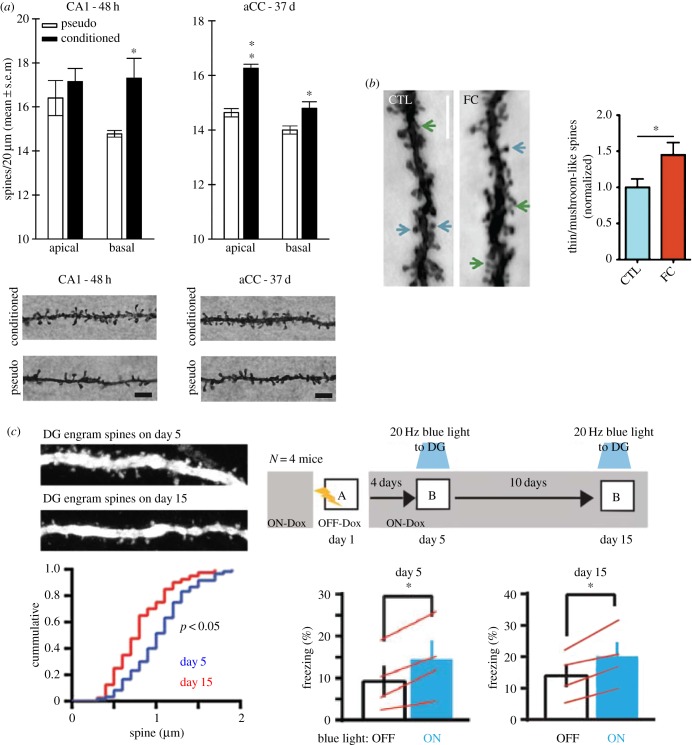
The relationship between structural synaptic plasticity and current theories on remote memory consolidation. (*a*) Findings of structural plasticity changes in alignment with the standard model of system consolidation. Recent memory elicits the formation of basal dendritic spines in hippocampal area CA1 while remote memory is associated with both apical and basal spine changes in the anterior cingulate cortex (aCC) ([55, fig. 4]). (*b*) Findings of structural plasticity changes in alignment with the multiple trace theory of memory consolidation. Spine density changes in anterior cingulate cortex occur within hours after contextual fear conditioning (FC) ([91, fig. 3]). (*c*) Local structural plasticity changes may not be needed for a memory to be accessible. DG engram-specific spine density at day 15 (remote memory) was significantly reduced compared with that on day 5 (recent memory), but on both days, optogenetic activation of DG engram cells induced behavioural freezing ([18, fig. 3]). All figures are reproduced with permission. (Online version in colour.)

In support of the classic system consolidation theory, Restivo *et al.* reported a time-dependent increase in spine density in the hippocampus for recent and in the ACC for remote memories, respectively (figure 2*a*) [55]. Furthermore, ibotenic hippocampal lesions immediately after conditioning, but not 24 days later, impaired remote memory and prevented dendritic spine growth in the ACC, emphasizing that the hippocampus is of crucial, but time-limited importance in driving structural plasticity in the cortex. Further, in accordance with the idea that cortical memories are expressed independently of the hippocampus at remote timepoints, Vetere *et al.* reported that when ACC spine growth at two different times (1 and 42 days) following contextual fear conditioning was locally disrupted using viral injections of the transcription factor MEF2, which negatively regulates spinogenesis, memory consolidation was impaired [121].

In contrast, another study found that the ratio of thin spines to mushroom spines in the mPFC was already significantly increased 1 h following contextual fear conditioning (figure 2*b*), supporting the idea of early cortical structural remodelling at the time of memory encoding [91]. Importantly, here, the formation of not only recent but also remote memories was impaired by a temporally precise optogenetic inhibition of excitatory mPFC neurons during conditioning. Although this study only provided snapshots of dendritic spines (using the Golgi–Cox impregnation method), it nevertheless emphasized the crucial role of an early involvement of mPFC spines in remote memories, akin to the MTT.

To complicate matters even further, a recent two-photon microendoscopy study monitored turnover dynamics of basal dendritic spines in hippocampal pyramidal neurons and found a near full erasure of the synaptic connectivity pattern within 15 days post-learning [54]. This finding is not only in stark contrast with cortical spine stability described above [55], but also stipulates that hippocampal spines may not be suited to support longer-lasting memories (mPFC spines were not monitored in this study), although spines are known to occur in an engram-specific manner [29]. In agreement with these ideas, a reduced number of spines in DG engram cells at 14 days post-training was recently reported, despite the fact that behavioural fear expression could be elicited optogenetically (figure 2*c*) [18]. Collectively, these incongruent findings point to the need for an alternative explanation to spine dynamics for remote memory stability.

## 5. Epigenetic modifications and remote memory

An interesting fact in memory consolidation is the observation that the longer-lasting it is, the more resistant it appears to disruption [122,123], giving rise to a consequential passage of time effect once a commitment for a permanent change has taken place. But what might this change be, if it is not at the level of the spine? Certainly, a change of lasting nature. As such, epigenetic mechanisms were postulated more than 30 years ago to explain the lifelong basis of a memory [124,125], and demonstrated for the first time to be implicated in LTP and memory formation 20 years after the original postulate [126–128]. Ever since, an increasing number of studies have pointed towards the importance of epigenetic mechanisms to resolve the short-lived nature of synaptic events associated with LTP, learning and memory, and the need for a self-perpetuating signal to preserve long-lasting memories [42–44,129,130].

Epigenetic mechanisms studied in the field of memory research are essentially of two types, namely DNA methylation [43], and post-translational modifications on histone tails [131]. DNA methylation, a mainly transcriptionally repressive transcriptional mechanism, is based on the covalent addition of methyl groups to cytosine bases in CG-rich stretches of the DNA, called CpG islands. Through gene-specific analysis of CpG islands in the promoter region of calcineurin and reelin, cortical DNA methylation has been found to not only accompany but also be necessary for memories lasting 30 days [132], while the hippocampus was characterized by only transient changes in the days following contextual fear conditioning [132,133]. More recently, another study reported increased DNA 5-hydroxy methylation levels, another type of DNA-based epigenetic modification, at the CpG-enriched coding region of the IEG *c-Fos*, but not of *Npas4*, for remote contextual fear memories, while both genes showed the same epigenetic modification for recent memories [134]. These studies point to the intriguing possibility of gene-specific DNA-based epigenetic modifications that are important for long-term memory maintenance.

In like manner, posttranslational histone modifications in terms of acetylation, phosphorylation and methylation have also been found to occur transiently (1 day) in the hippocampus, but more persistently (7 days) in the cortex, where they facilitated the consolidation of object memories [135]. Several other studies have confirmed a role for histone acetylation in long-term memories. Transgenic mice that express CREB binding protein with reduced histone acetyltransferase activity [127,128] failed to stabilize short-term into long-term memories, yet their behavioural phenotype was rescued by the administration of the histone deacetylase inhibitor (HDACi), Trichostatin A. HDACi-mediated increments in histone acetylation were further found to be associated with improved remote olfactory memory retrieval by infusing sodium butyrate (NaB) or Trichostatin A into the cortex in the first few days following learning, but not long (15 to 27 days) after acquisition [76], which supports that notion of a ‘priming’ capacity of epigenetic modifications [45]. In another recent study, HDACi promoted long-term memory but not short-term memory retention of spatial information immediately after a subthreshold spatial learning [136].

In addition to posttranslational histone modifications, histone exchange and turnover have recently also been implicated in memory consolidation. When H2A.Z, a variant of the core histone H2A, was depleted locally in hippocampus, both recent and remote memory testing were improved, in sharp contrast to its local depletion in mPFC, upon which freezing was higher at remote (30 days) testing only [137]. In similar fashion, when histone turnover with the H3 variant H3.3 was genetically prevented, both novel object recognition and contextual fear memory consolidation were reduced [138], testifying again to the implication of chromatin-based processes in long-term memory maintenance.

A major drawback of all but a few of these studies (e.g. [139]) is that epigenetic modifications continue to be investigated at the heterogeneous whole-tissue level. Notwithstanding, because these changes are indeed detectable at such a gross level, their implication in memory formation cannot be neglected. Future work analysing epigenetic modifications of different cell types, within dedicated circuits and in response to established memory-related signalling cascades, are likely to clarify this view.

## 6. Synopsis

In the present review, we have collected the available evidence on the current knowledge of remote memory consolidation. Important experimental data have surged in recent years on the relevance of cortical modules in remote memory formation beginning at its encoding state [18,76,83,91,92], a revolutionary concept that dares to break the dogma of labile memories shifting from hippocampus to neocortex at later stages as stated by standard theories of consolidation [51,140]. In addition, the importance of the hippocampus at retrieval, from recent to remote timepoints [2,93,141], is also more in agreement with the MTT than the standard consolidation model. Nevertheless, the unidirectional, irreversible hippocampal–neocortical transfer during consolidation continues to attract substantial attention given clinical data from patients with retrograde amnesia [142–147], although unequivocal experimental evidence in support of it is lacking (for a review, see [52]). One potential explanation for this apparent discrepancy might be that MTT requires a transformation of memories over time into schematic representations, but if memories are hippocampus-bound—such as episodic or contextual ones—then mnemonic signals can remain available in the hippocampus for longer periods of time and their retrieval is more readily available [16,17]. What is more, the vast majority of studies on memory consolidation—for practical and technical reasons—investigated discrete retrieval at two timepoints post-acquisition for recent and remote memories, which implies that no neural signature of the spatiotemporal dynamics of consolidation has been established yet. As a result, we do not precisely know the circuit or cellular mechanisms for this information transfer nor its molecular characteristics.

Since the biological marking for remote memories in cortex already occurs at the time of encoding [18,76,91], it is likely that a parallel, simultaneously occurring process along key anatomical structures determines the fate of the memory into its remote configuration. This idea emphasizes (1) a location-dependent process where molecular changes will take place along critical periods; (2) that the fate of a memory is determined at encoding (at least until recall); and (3) that genetic and molecular processes, rather than activity-dependent neuronal firing along circuits, are responsible for system consolidation over a longer time scale. But what could control the expression of critical genes in certain brain structures to give the specificity and temporal precision needed for this tagging process?

In our view, epigenetic changes can form part of both molecular-synaptic and circuit-system level consolidation: the former because epigenetic modifications can lastingly alter intracellular signalling cascades to influence excitability and thereby modify synaptic properties [44]; the latter because these changes may also occur simultaneously across brain regions [139] in order to favour stability or further modify mnemonic signals. These changes may thereby account not only for the molecular and temporal precision needed for a long-term storage task but also for accommodating plasticity driven by learning, as epigenetic modifications readily react to changing cellular environments [44]. Accordingly, once a remote memory is committed to its fate, it will take an enormous amount of energy to overcome its new entropic state, or in molecular terms, the activation of the molecular signal machinery in reverse order will be unlikely. This would for instance explain the known resistance to revert a remote memory into a labile state (e.g. [123]). In support of this epigenetic idea, remote fear memories have recently been shown to respond to HDACi treatment during reconsolidation making them more amenable for attenuation [148]. Cortical DNA hyper-methylation has also been proposed as a mechanism to account for long-lasting changes in ACC and remote memories [132]. However, how precisely learning triggers these epigenetic changes, how well they are conserved across brain regions, and how these changes are signalled to and from the nucleus to the synapse remain to a large extent still elusive.

Part of the inherent difficulty in making assertive conclusions on theories of remote memory consolidation derives from the different techniques and paradigms used by different laboratories, but also from our limited understanding of the dynamics of certain mnemonic processes under certain conditions, for instance how a natural stimulus-driven retrieval might differ from an optogenetic activation of the ensembles emerging from that experience [18,29,149,150]. Also, our knowledge of engrams as neuronal entities themselves is hitherto based on IEG-dependent labelling, itself likely the result of high levels of expression of the transcription factor CREB [36,57–59], and thus far from being all-inclusive. Indeed, owing to their very recent appearance on the centre stage of memory consolidation, we still know little about engrams in terms of (1) their afferent or efferent connectivity with other structures of the fear circuit, (2) their internal anatomical connectivity or functional nodes, (3) their regulatory cycle of activity, maintenance and disappearance, (4) their overlap with other neuronal non-fear local ensembles such as spatial hippocampal information/context ensembles [151,152], or (5) the prefrontal cortex ensembles for association with multimodal sensory information [153–156]. Research on cell type specificity, cell-to-cell interaction and long-range connectivity of circuit assemblies remains pivotal for understanding their long-term maintenance, as for instance astrocyte–neuronal metabolic induction was reported to be important for memory consolidation [157] and several types of interneurons important for engram sizing [26,158].

Furthermore, it is also sometimes difficult to compare experimental results within the same brain area owing to inexistent data on comparison across subfields, their functional internal connectivity or intrinsic physiologic firing patterns during remote memory, or simply the stereotaxic coordinates used during surgery. As a result thereof (or not), sometimes contradictory results within the same anatomical region have been reported by different laboratories, e.g. the strength of a memory associated with more stable CA3 engrams [38] versus DG engrams [29–31,149], BLA being a critical nodal point in consolidation coordinating remote memory retrieval [18] versus BLA patterns of reactivation fading off together with the hippocampal ones after recent stages [37], or retrosplenial cortex playing a critical role for storage of remote memories [37,80,99] versus being without apparent importance [18].

Finally, incomplete and sometimes contradicting results also originate from our current understanding of synaptic remodelling in the form of dendritic spines for remote memories. Whether the transfer of mnemonic information happens in the form of synaptic remodelling is to date unclear as evidence speaks both in favour of synaptic remodelling supporting remote fear memories [121], and against it [18]. In this respect, it might be of high interest to replicate the work on optical erasure of synaptic memory traces done in motor cortex [150] in the context of remote memories, for a more causal relationship.

In the meantime, while these issues are being addressed, more data generated and current techniques improved, our knowledge on remote memories is likely to continue to consolidate.

## Data accessibility

This article has no additional data.
